# Does the uterine microbiota affect the reproductive outcomes in women with recurrent implantation failures?

**DOI:** 10.1186/s12905-022-01750-w

**Published:** 2022-05-14

**Authors:** Lela K. Keburiya, Veronika Yu. Smolnikova, Tatiana V. Priputnevich, Vera V. Muravieva, Alexey B. Gordeev, Dmitry Yu. Trofimov, Ekaterina S. Shubina, Taisiya O. Kochetkova, Margarita S. Rogacheva, Elena A. Kalinina, Gennady T. Sukhikh

**Affiliations:** National Medical Research Center for Obstetrics, Gynecology and Perinatology named after Academician V.I. Kulakov, Ministry of Healthcare of the Russian Federation, 4 Oparina Street, Moscow, Russia 117997

**Keywords:** Infertility, Assisted reproductive technologies, Uterine microbiota, Metagenomic sequencing, Embryo implantation

## Abstract

**Background:**

Inefficiency of in vitro fertilization (IVF) programs can be caused by implantation failures. The uterine microbiota can influence the implantation process. However, it still remains unclear whether opportunistic microorganisms detected in the endometrium have a negative impact on the implantation success. The aim of our study was to evaluate the influence of the uterine microbiota on the embryo implantation success in patients undergoing assisted reproductive technologies.

**Methods:**

The study included 130 women diagnosed with infertility. The patients were divided into three groups: group I included women with the first IVF attempt (n = 39); group II included patients with recurrent implantation failure following embryo transfer with ovarian stimulation (n = 27); group III consisted of women with recurrent implantation failure following frozen-thawed embryo transfer (n = 64). We performed microbiological examination of the embryo transfer catheter which was removed from the uterine cavity after embryo transfer; cervical discharge of all the patients was studied as well. Thirty patients were selected for metagenomic sequencing.

**Results:**

The study showed that the uterine cavity is not free of microorganisms. A total of 44 species of microorganisms were detected: 26 species of opportunistic organisms and 18 species of commensals (14 species of lactobacilli and 4 species of bifidobacteria). Obligate anaerobic microorganisms and Gardnerella vaginalis were detected more frequently in group I compared to group III (strict anaerobes—15.4 and 1.6%; G. vaginalis—12.8 and 1.6%, respectively) (*p* < 0.05). However, this fact did not have a negative influence on the pregnancy rate: it was 51.3% in group I, it was 29.6% and 35.9% in women with recurrent implantation failures, respectively.

**Conclusion:**

Opportunistic microorganisms which were revealed in low or moderate titers (10^3^–10^5^ CFU/ml) in the uterine cavity and cervical canal did not affect the pregnancy rate in the women in the study groups. The microflora of the uterine cavity and cervical canal differed in qualitative composition in 87.9% of patients, therefore, we can suggest that the uterine cavity may form its own microbiota. The microbiota of the uterine cavity is characterized by fewer species diversity compared to the microbiota of the cervical canal.

## Background

Overcoming infertility in patients with recurrent implantation failures (RIF) is a highly topical issue in reproductive medicine. RIF—failure to achieve pregnancy after multiple IVF attempts [1]. A favorable outcome of assisted reproductive technologies (ART) programs depends on a coordinated dialogue between the receptive endometrium and a high-quality embryo [1, 2].

Uterine microbiota and its influence on implantation success have been the subject of recent research. Microbiota are a group of microorganisms which are present in a human biotope and coexist symbiotically with the host organism. Due to the intensive study of the human microbiome, the role of the uterine microbiota during embryo transfer has been much emphasized [3–9].

To date, it still remains unclear whether opportunistic microorganisms detected in the endometrium have a negative impact on implantation, what composition of the uterine microbiota is considered to be normal and which one is associated with dysbiosis having an adverse effect on implantation, and where is the line between the norm and pathology in quantitative and qualitative ratio [10–13].

Therefore, the aim of the study was to evaluate the effect of the uterine microbiota on the success of implantation, the course of early pregnancy, and rate of live births in women undergoing IVF.

## Methods

The study included 130 reproductive-aged patients who underwent treatment at the National Medical Research Center for Obstetrics, Gynecology and Perinatology, Moscow, Russia. All of them complained of infertility.

The participants of the study met the following inclusion criteria: age from 23 to 37 years, regular menstrual cycle, absence of endometrial pathology confirmed by the ultrasound assessment, transfer of good quality embryos.

The exclusion criteria were the presence of interstitial and/or subserous uterine fibroids measuring more than 4 cm which could deform the uterine cavity, stage III–IV endometriosis, intrauterine pathology (intrauterine septum, endometrial polyp, chronic endometritis), severe pathozoospermia of a partner.

The patients were divided into three groups: group I consisted of women with the first IVF attempt (n = 39); group II included patients with recurrent implantation failure (RIF) following embryo transfer with ovarian stimulation (n = 27); group III consisted of women with RIF following frozen-thawed embryo transfer (n = 64).

Microbiological examination of the cervical microbiota of all women was performed before embryo transfer to the uterine cavity. The vaginal part of the cervix was cleansed with a sterile cotton swab to exclude contamination of the cervical canal by the vaginal microflora. The material was collected from the cervical canal using Dacron swab and placed into test tubes with Ames transport medium (Medical Wire, Great Britain). The composition of the microbiota was studied using culturomics and with a wide variety of selective and non-selective culture media. Cervical canal discharge was inoculated for selective and non-selective agar media. After embryo transfer into the uterine cavity, the distal fragment of the embryo catheter was cut off with sterile scissors and placed in a test tube with culture media (1 ml) used for hemocultures (Oxoid, Great Britain). After 48 h of cultivation in an anaerobic box (Jouan, France) filled with a three-component gas mixture (N_2_-80%; CO_2_-10%; H_2_-10%), the composition of the microbiota was identified by culturomics.

Isolated microorganisms were identified using time-of-flight mass spectrometry (MALDI-TOF MS) with an Autoflex II mass spectrometer and Maldi BioTyper software, version 3.0 (Bruker Daltoniks, Germany).

All women had a selective single embryo transfer. Patients of groups I and II underwent ovarian hyperstimulation with gonadotropin-releasing hormone (GnRH) antagonists from days 2–3 of the menstrual cycle. Standard dose of human chorionic gonadotropin (10,000 IU) was used as an ovulation trigger.

Frozen-thawed embryo transfer was carried out in a natural ovulatory cycle without hormone replacement therapy. After embryo transfer, dydrogesterone (30 mg/day) was administered orally from the day of transvaginal puncture of the ovaries in hyperstimulation cycle. There was a comparative analysis of the results obtained in women both in the ovarian hyperstimulation cycles and in frozen-thawed oocyte cycles, depending on pregnancy or its absence.

At the second stage of the study, 30 samples of biomaterial from the uterine cavity (a fragment of an embryo transfer catheter in the culture medium) were selected for next-generation sequencing with a subsequent comparison with microbiological data.

Variable regions V3-V4 were chosen to determine the species composition of the uterine microbiota using next-generation sequencing. Amplification of the selected region was performed with primers 357F and 806R; DNA extraction from samples was performed using PREP-RAPID DNA Extraction Kit, and amplification of the 16S rRNA gene sequence was carried out using the DTprime detecting thermocycler (“DNA-Technology, Research & Production”, LLC, Russia). The quality of the obtained amplicons was evaluated in a 2% agarose gel. The quality and concentration of DNA libraries for next-generation sequencing were checked using a bioanalyzer device. Sequencing was performed using the Illumina MiSeq system (USA) v2 kits according to the manufacturer’s protocol. Data analysis was conducted using the QIMME software package.

## Results

We performed a quantitative and qualitative analysis of the composition of the cervical canal microflora in women of the study groups.

**Fig. 1:**
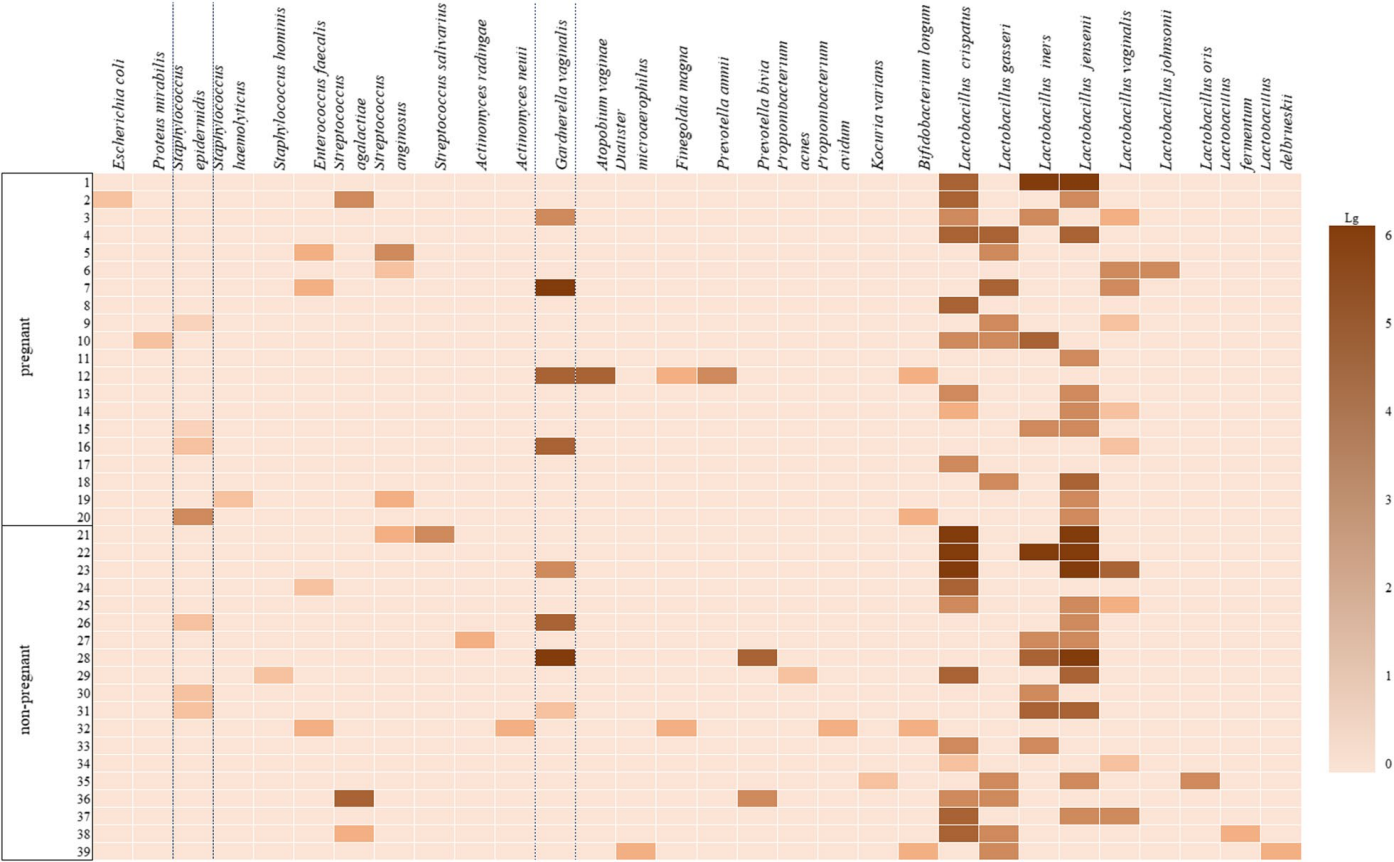
Quantitative evaluation of microorganisms in patients with the first IVF attempt

We revealed 30 species of opportunistic microorganisms in patients with the first IVF attempt, namely, 20 species of opportunistic microorganisms and 10 species of commensals. Lactobacilli were most often detected in moderate (10^4^–10^5^ CFU/ml) or large (10^6^ CFU/ml or more) amounts more often in women who became pregnant than in those who did not conceive (90.0 and 73.7%, respectively), but there was no statistically significant difference (*p* > 0.05). Opportunistic microorganisms were identified mainly in low titers (10^3^ CFU/ml or less). The exceptions were *Gardnerella* and obligate anaerobic microorganisms. For example, moderate or high titers of *G. vaginalis* were mainly detected in pregnant women (moderate titers—15%, high titers—5%) and in women who did not become pregnant (high titers—5.3%, moderate titers—10.6%). On the contrary, obligate anaerobic microorganisms were detected more frequently in low (15.8%) or moderate titers (10.5%) in women who did not conceive; pregnant women showed the same level of low and moderate titers (5% each), but there was no statistically significant difference either. Other opportunistic microorganisms, such as enterococci, enterobacteria and actinomycetes, colonized the cervical canal only in low concentrations. The level of microbial colonization taking into account the frequency of occurrence and quantitative assessment of microorganisms in women of group I is shown in Fig. 1.

The patients of group I who were pregnant or did not become pregnant showed no statistically significant difference in the frequency of detection of lactobacilli and opportunistic microorganisms in low, moderate or high concentrations in the cervical canal. Frequent colonization with lactobacilli in moderate or high concentrations, as well as frequent detection of opportunistic microorganisms (*G. vaginalis*) in moderate or high titers in patients of group I who became pregnant, did not significantly affect the implantation of the embryo.

**Fig. 2:**
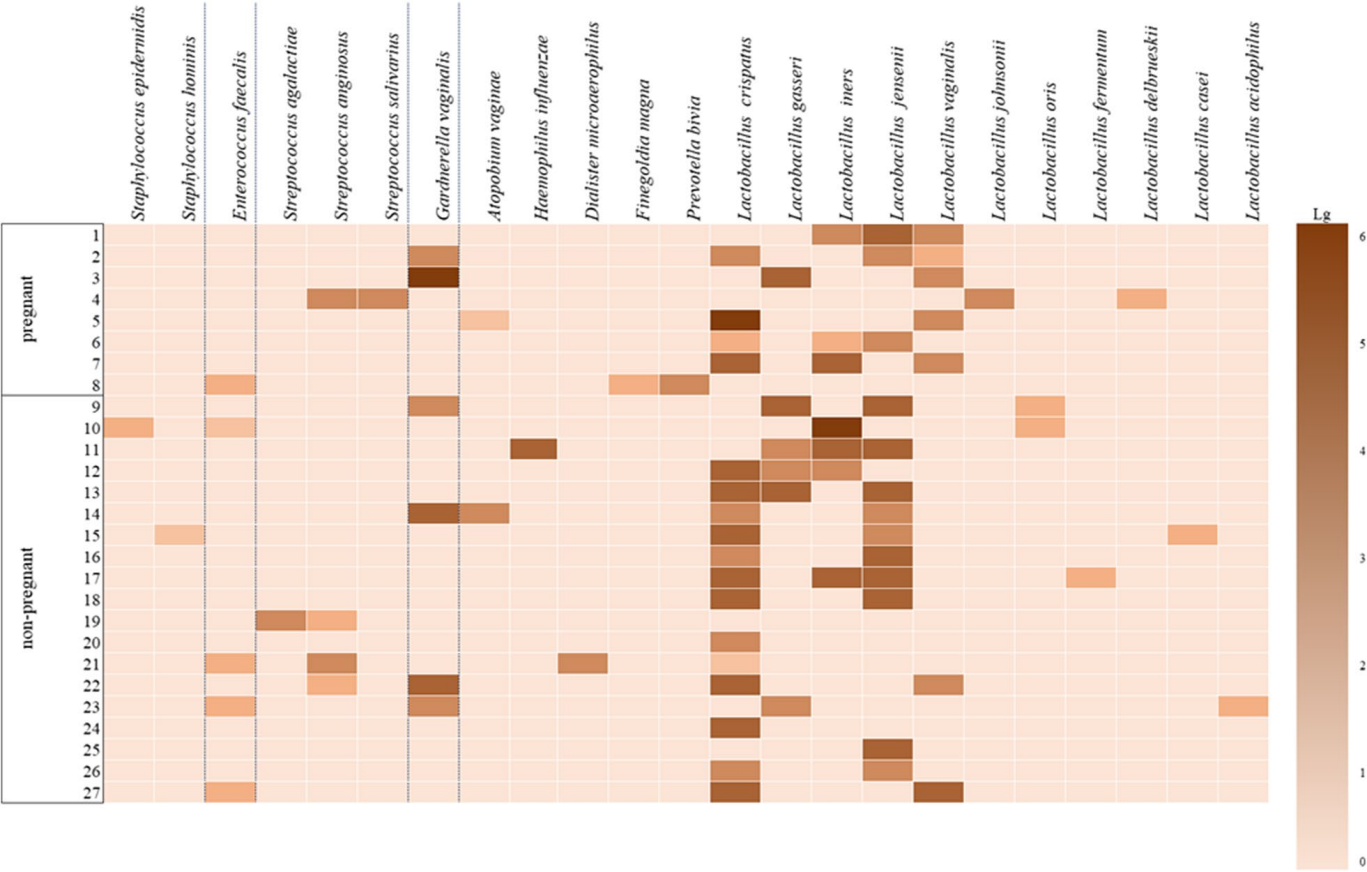
Quantitative evaluation of microorganisms in patients with RIF following embryo transfer and ovarian stimulation

Among 23 species of microorganisms revealed in women with RIF following embryo transfer with ovarian stimulation (group II), there were 12 species of opportunistic microorganisms and 11 species of commensals, lactobacilli. Lactobacilli in moderate or large amounts were more often detected in women who did not become pregnant than in pregnant women (63.2 and 50.0%, respectively) in patients of group II compared to patients of group I, but the results were not statistically significant (*p* > 0.05). Opportunistic microorganisms were cultured in low, moderate or high concentrations. Therefore, enterococci and coagulase-negative staphylococci were detected only in low concentrations; streptococci in both subgroups were cultured mainly in moderate concentrations and dominated in the group of pregnant women (12.5 and 5.3%, respectively). *Gardnerella* was found in both subgroups in moderate concentrations more often in women who were not pregnant (21.1%), and in high concentration in one pregnant woman (12.5%); strict anaerobes in low and moderate titers were also isolated more frequently in pregnant women (12.5 and 10.5%, respectively). The level of microbial colonization taking into account the frequency of occurrence and quantitative evaluation of microorganisms in women of group II is shown in Fig. 2.

The patients of group II who had RIF following embryo transfer with ovarian stimulation showed a lower frequency of detected lactobacilli, a lower degree of their contamination of the cervical canal and more frequent colonization of the cervical canal by opportunistic microorganisms (obligate anaerobes) in low and moderate concentration. However, there was no negative influence on the implantation and we may suggest that the presence of microorganisms in the cervical canal, which is anatomically as close as possible to the uterine cavity, in low and moderate concentrations does not affect the implantation and the beginning of pregnancy.

A total of 42 species were identified in patients with RIF following frozen-thawed embryo transfer (group III), namely, 25 species of opportunistic microorganisms and 15 species of commensals, 2 species of yeast fungi. Lactobacilli were most often revealed in moderate or large concentrations in women of group III, more frequently in women who had pregnancy than in women who did not become pregnant (60.9% and 51.2%, respectively), but no statistically significant difference was found (*p* > 0.05). Opportunistic microorganisms were detected mainly in low concentrations, except *Gardnerella* which was found in both subgroups in moderate concentrations (4.3% and 7.3%, respectively). Strict anaerobes were detected in pregnant women only in low concentrations (13.0%), women who did not conceive showed the same frequency in low and moderate titers (9.75%). Coagulase-negative staphylococci and enterococci were almost always present in low concentrations.

**Fig. 3:**
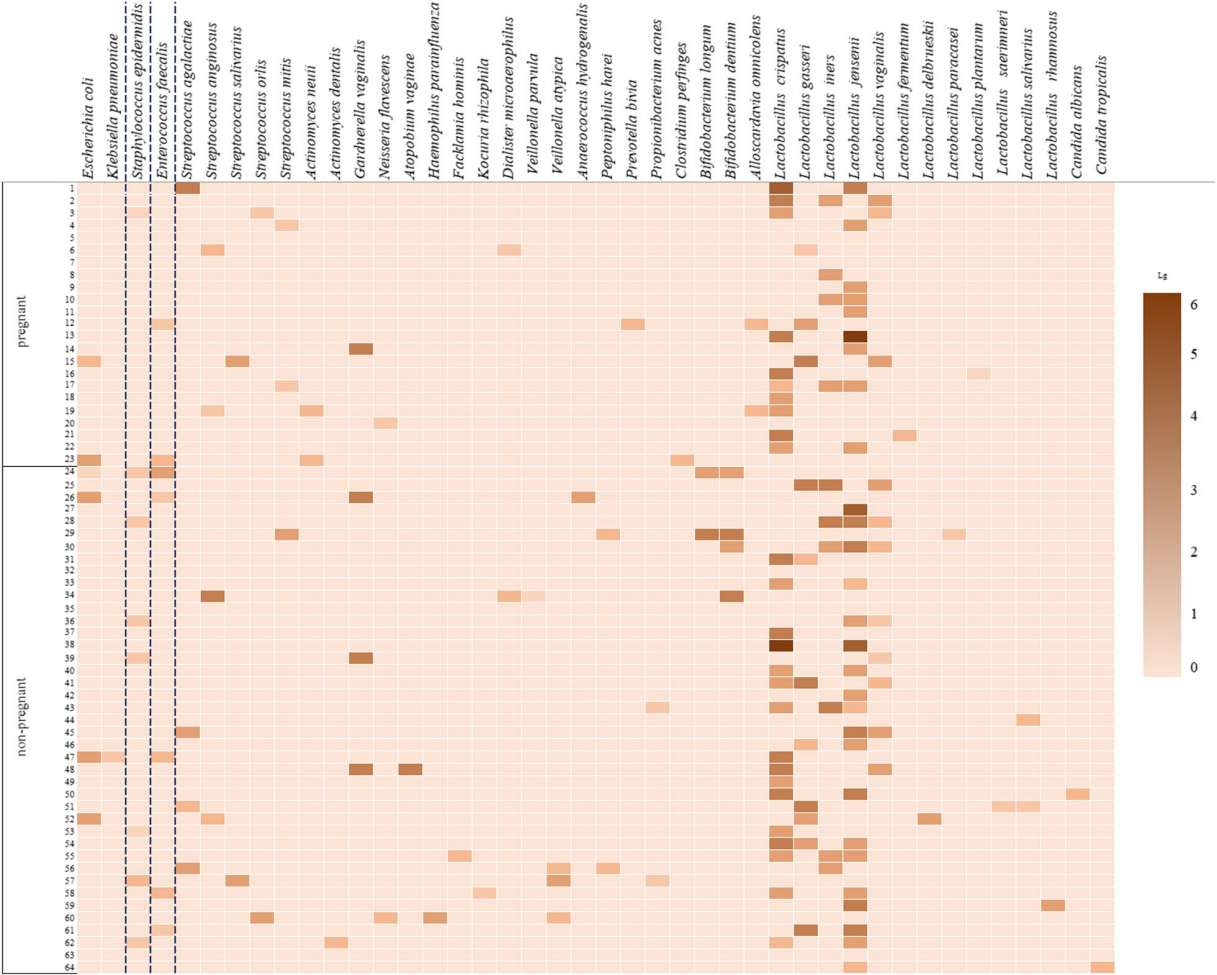
Quantitative evaluation of microorganisms in patients with RIF following frozen-thawed embryo transfer

There was no statistically significant difference in the frequency of colonization of the cervical canal by the microorganisms in low and, what is more important, in moderate concentrations between pregnant and non-pregnant women (*p* > 0.05). The level of microbial colonization taking into account the frequency of occurrence and quantitative evaluation of microorganisms in women of group III is shown in Fig. 3.

The patients with RIF following frozen-thawed embryo transfer who did not conceive were less likely to have colonization of the cervical canal with lactobacilli in high or moderate concentrations than those who became pregnant. On the contrary, opportunistic microorganisms (streptococci, enterobacteria, and especially strict anaerobes and *Gardnerella*) were revealed in moderate concentrations. However, due to the absence of a statistically significant difference in these indicators, we cannot consider the decrease in the titer of lactobacilli, as well as the increase in the number of opportunistic microorganisms to moderate values as a risk factor for implantation failure.

After the comparative analysis of the cervical canal microorganisms in women of the study groups, we found that the most common microorganisms of the cervical canal in all groups of women were lactobacilli, which were slightly more frequent in women with embryo transfer with ovarian stimulation (groups I and II) compared to women with frozen-thawed embryo transfer (group III), but statistically significant difference was not noted. Two species of lactobacilli, *L. jensenii* and *L. crispatus*, prevailed in the frequency of colonization in all groups, but women of groups I and II had a certain predominance (*p* > 0.05). The cervical canal was colonized only by lactobacilli in almost 40.0% of women of all groups (33.3%; 44.4% and 36.0%, respectively), mainly by associations of two or three species. Opportunistic microorganisms were found in every second woman in all groups (with a slight predominance in group I). In all groups, there was no statistically significant difference in the colonization of the cervical canal with facultative and obligate anaerobes. *G. vaginalis* was revealed more frequently in women of groups I (20.5%) and II (22.2%) compared to group III (6.3%), but the difference was statistically insignificant (*p* > 0.05).

**Fig. 4:**
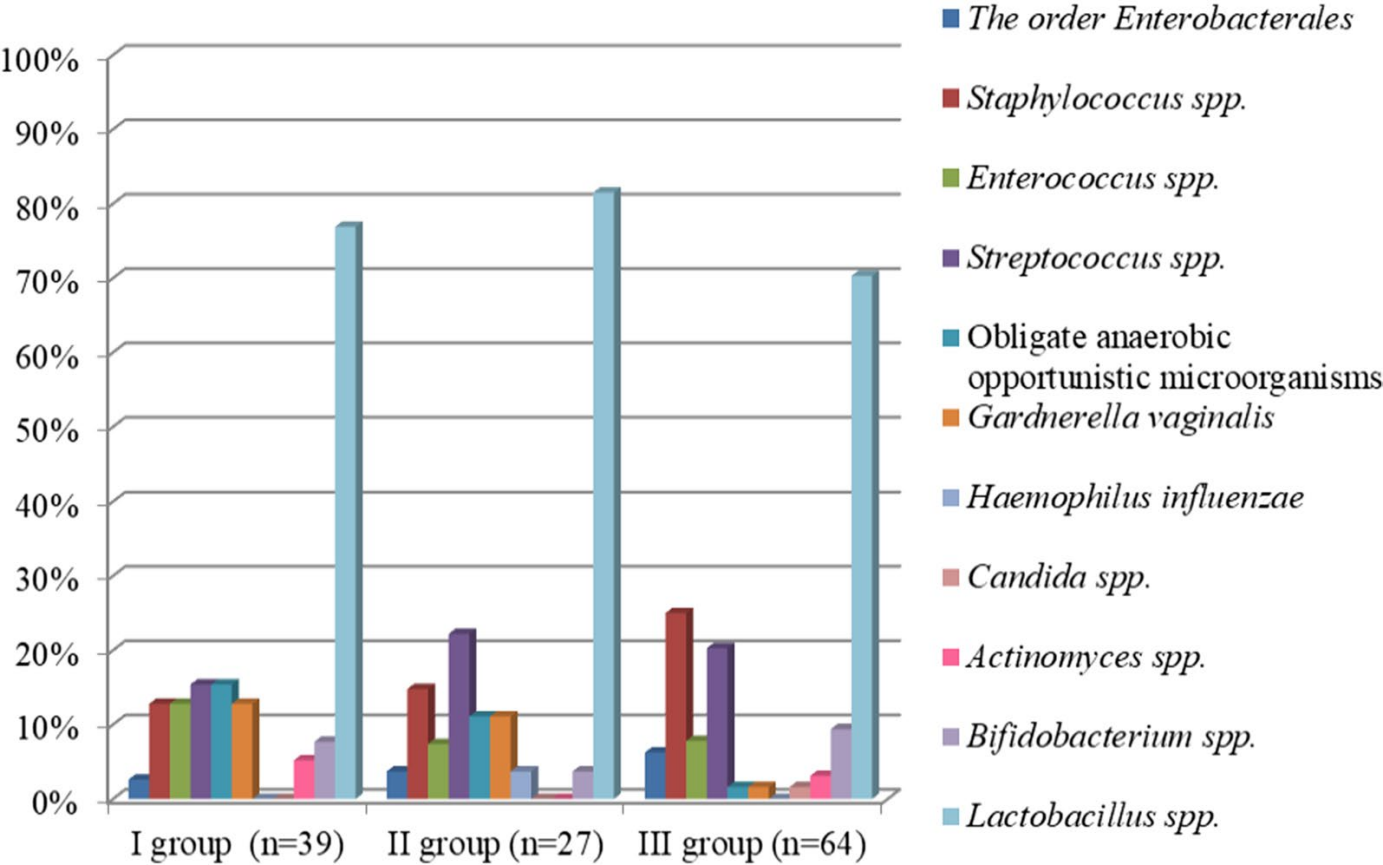
Comparative analysis of the frequency of detecting microorganisms in the uterine cavity in study groups. *Note* I–III *G. vaginalis* (*p* = 0.05); I–III obligate anaerobic opportunistic microorganisms (*p* = 0.02)

Uterine cavity aspirate of the patients was analyzed as well. The results of a comparative analysis of the uterine microbiota in the groups of women are shown in Fig. 4.

Culture-based analysis of the content of the uterine cavity in patients of all three groups showed the presence of microflora in 89.2% of cases. The uterine cavity in women of groups I, II and III was found to be sterile only in 10.3%, 7.4% and 12.5% of cases, respectively. The species of microorganisms isolated from the uterine cavity were diverse. Lactobacilli (14 species) were dominant in all groups, three of them were more frequent, namely *L. jensenii*, *L. crispatus* and *L. vaginalis*. The frequency of isolation of *L. jensenii* in the study groups was 48.7%, 51.9% and 40.6%, respectively. The isolation rate of *L. crispatus* was 35.9%, 25.9% and 31.3%, respectively. The frequency of occurrence of *L. vaginalis* in women of the study groups was 2.6%, 11.1% and 9.4%. There was no statistically significant difference in the frequency of colonization of the uterine cavity by lactobacilli and in isolation rate of individual types among patients of the study groups (*p* > 0.05). Isolation rate of opportunistic microorganisms was the same in patients of groups I, II, and III (46.1, 44.4 and 48.4%, respectively (*p* > 0.05)).

Enterobacteria were detected more often in pregnant women with RIF following frozen-thawed embryo transfer (group III) (17.4%) compared to the women who became pregnant after the first IVF attempt (group I) (5%) and women with RIF following embryo transfer with ovarian stimulation (group II) (12.5%). Staphylococci were detected more frequently in patients of group III (25.0%) in comparison with patients of group I (12.9%) and group II (14.8%). Streptococci were revealed more frequently in patients with RIF in groups II (22.2%) and III (20.3%) compared to women of group I with IVF attempt (15.4%). It is worth noting that isolation rate of obligate anaerobic microorganisms in women with the first IVF attempt was 15.4% compared to patients with RIF following frozen-thawed embryo transfer whose rate was 1.6%. The frequency of detection of *G. vaginalis* in women with the first IVF attempt was 12.8% and it was 1.6% in women with RIF following frozen-thawed embryo transfer. Thus, isolation rate of obligate anaerobic microorganisms and *G. vaginalis* was statistically significantly higher in group I compared to group III (*p* = 0.02, *p* = 0.05, respectively).

Metagenomic study of 30 samples of uterine cavity aspirate was carried out. The most common bacterial genera (the occurrence is higher than 5.0%) are presented according to the sequencing of a fragment of the 16S ribosomal RNA gene.

An extremely low concentration of bacterial DNA was found in all samples of the sterile uterine cavity compared to the concentration of DNA in negative control samples. Microorganisms of the genera *Lactobacillus, Tepidimonas, Streptococcus, Proteus, Acinetobacter, Enterococcus, Pseudomonas, Veillonella* were detected in these samples; such genera were also detected in negative control samples.

In most cases, the data on representation of microorganisms in the uterine cavity obtained after microbiological studies were consistent with the data obtained after sequencing. In some cases, microbiological studies failed to detect some bacterial genera that were revealed by sequencing. These results can be explained by the presence of microorganisms which did not grow on the culture medium for various reasons, however their DNA was present in the sample.

The quantitative ratio of microorganisms was determined by sequencing, but these data should be considered carefully, since we studied the samples of the culture medium, but not the sample itself, and the ratio may depend on the growth rate of various microorganisms.

We conducted a comparative analysis of the microbiota of the uterine cavity and the cervical canal of 130 women. The analysis showed that the microbiota of the uterine cavity and the cervical canal was identical only in 14 (12.1%) of 116 women, and it was different in qualitative composition in 102 (87.9%) patients. When comparing the isolation rate of various components of the microbiota of the uterine cavity and the cervical canal, it was noted that a statistically significant difference was observed in the isolation rate of *G. vaginalis* (*p* = 0.01) and the most common lactobacillus species: *L. crispatus* (*p* = 0.01), *L. gasseri* (*p* = 0.01), *L. vaginalis* (*p* = 0.01) and *L. iners* (*p* = 0.01). As for the other species, the difference was not statistically significant (*p* > 0.05).

We analyzed the reproductive outcomes in women of the study groups (Table 1).

**Table 1:** Characteristics of pregnancy outcomes in patients of the study groups

Parameter	Group I (*n* = 39)	Group II (*n* = 27)	Group III (*n* = 64)
Pregnancy rate	51.3% (*n* = 20)	29.6% (*n* = 8)	35.9% (*n* = 23)
Biochemical pregnancy	5.0% (*n* = 1)	0% (*n* = 0)	4.3% (*n* = 1)
Missed abortion	0% (*n* = 0)	12.5% (*n* = 1)	17.4% (*n* = 4)
Spontaneous miscarriage	10.0% (*n* = 2)	0% (*n* = 0)	4.3% (*n* = 1)
Ectopic pregnancy	5.0% (*n* = 1)	0% (*n* = 0)	0% (*n* = 0)
Preterm birth	5.0% (*n* = 1)	12.5% (*n* = 1)	0% (*n* = 0)
Full-term delivery	75.0% (*n* = 15)	75.0% (*n* = 6)	73.9% (*n* = 17)

Group I included patients with the first IVF attempt (n = 39); group II consisted of women with recurrent implantation failure following embryo transfer with ovarian stimulation (n = 27); group III included patients with recurrent implantation failure following frozen-thawed embryo transfer (n = 64)

According to the data presented in Table 1, the ratio of pregnancy rate to embryo transfer in patients of group I was 51.3% and it was 1.7 times higher than in women of group II (29.6%) and 1.4 times higher than in women of group III (35.9%), but the difference was not statistically significant (*p* > 0.05). The analysis of pregnancy outcomes in the study groups showed the absence of missed abortions in patients with the first IVF attempt (0%), but this indicator was 12.5% and 17.4% in patients with RIF, groups II and III, respectively. However, statistically significant difference was not found (*p* > 0.05). The ratio of full–time deliveries to the number of pregnancies was 75%, 75% and 73.9% in the groups, respectively.

## Discussion

The importance of uterine microbiota in embryo transfer has recently received a lot of attention in reproductive medicine. There is no consensus regarding the sterility of the uterine cavity. And there is still no clear understanding of the influence of the endometrial microflora on the implantation processes. Moreover, it is not obvious what refers to eubiotic state of microbial ecology and what is dysbiotic disorder, as there is no clear line between these two concepts. The detection of opportunistic microorganisms in the reproductive tract and antibacterial therapy frequently fail to result in success [14–16].

The results of our study demonstrated that 87.7% of women had uterine microflora. Microbial communities can be found in the uterine cavity and the results of our study are consistent with the results of other authors [17–24].

The research conducted by Moreno et al. [17] demonstrated the influence of the uterine microbiota on implantation. It was revealed that the group of patients with a decreased percentage of *Lactobacilli* (< 90%) and prevalent opportunistic microorganisms (> 10%) in the uterine cavity in comparison with the group with prevalent *Lactobacilli* (> 90%) had a significantly lower implantation rate (60.7% vs. 23.1%, *p* = 0.02), pregnancy rate (70.6% vs. 33.3%, *p* = 0.03), progressing pregnancy (58.8% vs. 13.3%, *p* = 0.02) and birth rate (58.8% vs. 6.7%, *p* = 0.02).

The study of Kyono [25, 26] presented the analysis of the pregnancy rate in patients with prevalent *Lactobacilli* (*Lactobacilli* > 80%) and in patients without dominance of *Lactobacilli* (*Lactobacilli* < 80% and opportunistic microorganisms > 20%) in the uterine cavity. Pregnancy rate was higher in women with prevalent *Lactobacilli* (> 80%) compared to patients without dominant *Lactobacilli* (< 80%), 61.3% vs. 40%, respectively.

The opposite evidence is provided by the studies of other authors. Kotaro et al. [27] showed that the predominance of *Lactobacilli* in the uterine cavity (> 90%) was observed more often in women with RIF—64.3% (18/28) than in the control group—38.9% (7/18). The analysis of the vaginal samples showed similar results: *Lactobacilli* dominated in women with RIF—67.9% (19/28), unlike patients with the first IVF attempt—44.4% (8/18). Isolation rate of Gardnerella was 39.3% (11/28) in women with RIF and 27.7% (5/18) in the control group.

Thus, the researchers tend to focus on pathogenetically significant interaction between the endometrial microbiota and its immunity, and not just confirmation of the presence of microorganisms in the endometrium.

The results of our study are consistent with the results of other researchers [23–26]. There is no clear evidence that the presence of dominant *Lactobacilli* in the uterine cavity are beneficial in terms of pregnancy outcomes. But the restoration of the uterine microbiota with the aim of the predominance of *Lactobacilli* can have a positive effect on embryo implantation. The presence of opportunistic microorganisms in the uterine cavity did not affect the pregnancy rate.

Probably, uterine microbiota is a set of functionally associated microorganisms [24]. Certain microorganisms of the uterine cavity obviously support its homeostasis in every healthy individual. Biofilms, which are microbial communities, play an important role. Bacteria in biofilms have certain physiological properties. Normal biofilms in the human body are represented by microbial communities that form the physiological microflora of the skin, oral cavity, vagina, intestines, etc. But there are also pathological biofilms, which are often associated with chronic inflammatory processes.

The qualitative composition of microbiota of the uterine cavity and cervical canal was different in 87.9% of patients, therefore, one cannot exclude the formation of an independent microbiota in the uterine cavity, which is characterized by less species diversity compared to the cervical canal.

There is still no clear opinion about the composition of the microbiota of the upper and lower genital tracts. Moreno et al. found that microbial composition of the vagina and endometrium was different in 20% of the examined samples [17, 20]. Differences between these biotopes were found by Wee et al., who compared the uterine microbiota with vaginal and cervical samples in fertile and infertile women [28]. These results indicate that the microbiota of the upper and lower parts of the reproductive tract may be similar, but not always identical.

A separate stage of the study was the metagenomic sequencing of the uterine microbiota (the distal part of the embryo transfer catheter in the culture medium). This method of studying microbiota is described in the studies of some researchers [21, 22]. In most cases, the microorganism diversity in the uterine cavity was the same according to microbiological studies and metagenomic analysis. In some cases, there were bacterial genera that were not identified with microbiological studies. The results can be explained by the presence of microorganisms in the sample; these microorganisms did not grow in the culture media for various reasons, but their DNA was present in the sample. The obtained data may be due to the presence of trace amounts of bacterial DNA in the reagents and indicate that sequencing method is not sufficiently specific for analyzing samples with an extremely low DNA concentration. It can be assumed that besides the presence of microorganisms in the uterine cavity, a quantitative assessment of the composition of the microflora is important; however, it can be problematic to carry out the assessment at the time of embryo transfer due to insufficient amount of material obtained with an embryo transfer catheter.

Therefore, the use of this method for assessing the microflora of the uterine cavity at the time of embryo transfer remains questionable. The use of sequencing may be appropriate for a comprehensive assessment of the endometrial microbiota and its receptivity when obtaining biological material using a pipelle biopsy, as the material has a larger biomass than in case of embryo transfer catheter.

The condition of the whole organism is also essential since the endometrium can be protected against the destructive effect of the microbial factor. Microorganisms are known to be inactivated by cells involved in innate and adaptive immunity responses under physiological conditions [29]. In the early stages, this process is carried out by Toll-, NOD-, and RIG-like receptors which are located on the surface of cells of the immune system, epithelial cells, endothelial cells, and fibroblasts [29]. Microbial colonization of the endometrium is also restrained by Toll-like receptors, which stimulate the mechanisms of innate antimicrobial resistance by interacting with the structures of the microbial cell [30].

We analyzed the reproductive outcomes in women included in the study. The ratio of pregnancy rate to embryo transfer in patients of group I was maximal (51.3%), compared to the patients with RIF (29.6% and 35.9%, respectively). In spite of the fact that isolation rate of obligate anaerobic microorganisms and *G. vaginalis* was statistically significantly higher in group I compared to group III (strict anaerobes—15.4 and 1.6%; *G. vaginalis—*12.8 and 1.6%, respectively) (*p* = 0.02; *p* = 0.05), it did not negatively influence the implantation process in this group of women. The rate of missed abortions was 12.5% and 17.4% in patients with RIF (groups II and III, respectively), but there were no cases of missed abortions among women with the first IVF attempt. The ratio of full–term deliveries to the number of pregnancies was 75%, 75% and 73.9% in the groups, respectively.

## Conclusion

The most common microorganisms in the composition of the microbiota of the uterine cavity were lactobacilli in women who became pregnant and who were not pregnant: lactobacilli were 80.0% and 73.7% in group I, respectively; 90.2% and 79.0% in group II, and 60.9% and 75.6% in group III. The results of the study do not support the hypothesis about the role of lactobacilli and non-lactobacilli microbiota in the genesis of embryo implantation failures.

The comparative data on the composition of the microbiota of the uterine cavity and the cervical canal confirm the concept of non-sterility of the uterus and the existence of microbiota which is different from the microbiota of the lower parts of the reproductive tract. The qualitative composition and isolation rate of various types of microorganisms found in the uterus and cervical canal in the general cohort of women were identical only in 12.1% of women, and they differed in 87.9% of women.

The isolation rate of opportunistic microorganisms from the uterine cavity and the cervical canal, as well as the quantitative composition of their detection in the cervical canal in moderate concentrations (up to 10^5^ CFU/ml) among pregnant and non-pregnant women did not have statistically significant differences and did not affect the outcomes of ART in the study groups.

Therefore, taking into account the lack of statistical data confirming the influence of certain uterine microorganisms on the reproductive outcome, it is necessary to continue studying uterine microbiota. It is important to consider not only the present microorganisms, but also the individual characteristics of the whole organism, as well as the interaction of microorganisms with the organism. A clear understanding of the mechanisms of dysbiotic disorders leading to chronic inflammatory processes in the uterine cavity, as well as its timely diagnosis and treatment, is one of the fundamental issues in the treatment of patients with a history of implantation failures.

## Data Availability

All data generated or analyzed during this study are included in this published article.

## Abbreviations

IVF: In vitro fertilization
RIF: Recurrent implantation failure
ART: Assisted reproductive technologies
CFU: Colony forming units
GnRH: Gonadotropin-releasing hormone
