# Correction to: Does the uterine microbiota affect the reproductive outcomes in women with recurrent implantation failures?

**DOI:** 10.1186/s12905-023-02302-6

**Published:** 2023-05-15

**Authors:** Lela K. Keburiya, Veronika Yu. Smolnikova, Tatiana V. Priputnevich, Vera V. Muravieva, Alexey B. Gordeev, Dmitry Yu. Trofimov, Ekaterina S. Shubina, Taisiya O. Kochetkova, Margarita S. Rogacheva, Elena A. Kalinina, Gennady T. Sukhikh

**Affiliations:** National Medical Research Center for Obstetrics, Gynecology and Perinatology named after Academician V.I. Kulakov, Ministry of Healthcare of the Russian Federation, 4 Oparina Street, 117997 Moscow, Russia

Correction: BMC Women’s Health (2022) 22:1

10.1186/s12905-022-01750-w

Following publication of the original article [1], the reference no. 23 updated and the same has been shown below:

Einenkel R, Zygmunt M, Muzzio DO. Microorganisms in the healthy upper reproductive tract: from denial to benefcial assignments for reproductive biology. Reprod Biol 2019;19(2):113–118. 10.1016/j.repbio.2019.04.001. Epub 2019 Apr 22. PMID: 31023521.

The original article has been corrected.
